# Measurement of Personal Experienced Temperature Variations in Rural Households Using Wearable Monitors: A Pilot Study

**DOI:** 10.3390/ijerph17186761

**Published:** 2020-09-16

**Authors:** Rongjiang Ma, Yu Fu, Mengsi Deng, Xingli Ding, Jill Baumgartner, Ming Shan, Xudong Yang

**Affiliations:** 1 Department of Building Science, Tsinghua University, Beijing 100084, China; marj_2015@tsinghua.org.cn (R.M.); fu-y15@tsinghua.org.cn (Y.F.); dms15@tsinghua.org.cn (M.D.); dingxinglili@163.com (X.D.); xyang@tsinghua.edu.cn (X.Y.); 2 Institute for Health and Social Policy and Department of Epidemiology, Biostatistics and Occupational Health, McGill University, Montréal, QC H3A 1A3, Canada; jill.baumgartner@mcgill.ca; 3 China Association of Building Energy Efficiency, Beijing 100831, China

**Keywords:** personal thermal environment experience, wearable monitors, personal experienced temperature, rural environment

## Abstract

The time-varying data of air temperatures experienced by people in their daily lives is an important basis for studying human thermal sensation, adaptation, comfort, and health. It is also very important for designing targeted strategies to help people reduce uncomfortable experience. In this study, a small (98 mm × 49 mm × 25 mm), lightweight (~100 g), and portable temperature logger with a wide measurement range (−40 to 100 °C) and appropriate accuracy (±0.3 °C precision) was combined with a phone holder that was adapted as an armband sleeve to constitute a wearable monitor. Fourteen monitors were worn by 14 residents in 6 different households in rural Beijing, China, to monitor their personal thermal environment. In the context of having very similar living habits in winter and coping strategies for thermal discomfort, the temperatures that 14 residents experienced exhibited wide ranges and large variations during the two-day test period. The highest and lowest temperatures experienced by residents reached 30.6 and −16.6 °C, respectively. This paper provided new data and evidences about various temperatures experienced by residents, even though they were from the same family and lived together for decades. In terms of methodology, as an exploration, the present study indicated that using personal wearable monitors is a viable method to capture the real experienced thermal environment, which extended the method for collecting data regarding complex experiences in different environments to aid the study of human responses to the real-world thermal environment.

## 1. Introduction

In recent years, the Chinese government has promoted a clean energy for winter heating action in northern China aimed at reducing air pollution from traditional coal-fired heating and improving heating comfort [1]. In this process, there is a lack of clear basis for indoor comfort design parameters for heating in rural areas [2]. Our research team has carried out a series of related work, including understanding the different heating behaviors and actual heating demands of rural residents from the perspective of heating equipment [3] and different functional rooms [4]. Building on these studies, it is a worthwhile endeavor to further investigate the issue from the perspective of residents, by continuously monitoring the thermal environment they experienced, in the hope of seeking out the personal factors that lead to different heating demands on the one hand, and on the other hand, to find out the residents’ personal uncomfortable experiences and to help them reduce these experiences at a later stage by, for example, enhancing heating or minimizing overheating during the corresponding experience periods. However, lots of studies have focused on proposing generalized thermal neutral temperatures for rural residential buildings in the heating season, such as 18.4 °C in Beijing [5], 15.1 °C in the Northwest China [6], and 14.4 °C in the Northeast China [7], through field testing of indoor environments and questionnaire surveys. Others compared and evaluated the thermal neutral temperature and indoor thermal comfort in different regions in winter [8,9]. Although studies have shown that outdoor temperature affects indoor temperature and thermal comfort during the heating season [10,11], and only few studies have recorded the number of outdoor activities and the proportion of time spent outdoors manually [12], or recorded residents’ yard activity with video camera [13]. These studies however failed to link residents’ experiences in different thermal environments in an unbroken sequence; thus, they blocked the examination of their thermal comfort from the perspective of continuous exposure of the human in different environments. Nevertheless, most existing studies of the thermal environment and thermal comfort are based on steady-state conditions [14,15,16,17]. Unsteady conditions often occur in the real world in the form of temperature cycles, temperature drifts or ramps, and transients [18]. When ASHRAE (American Society of Heating, Refrigerating and Air-Conditioning Engineers) Standard 55 revised in 2004, it specified for the first time acceptable operative temperature ranges for naturally conditioned spaces based on an adaptive model of thermal comfort, which is considered to be the comfort requirements for unsteady conditions [19]. However, it has a strictly limited scope of application. For example, it is only suitable for the spaces where the habitants are engaged in near sedentary activities with metabolic rates ranging from 1.0 to 1.3 met [20]. In the 2005 revisions to ISO (International Organization for Standardization) Standard 7730, although the quantitative means for evaluating unsteady conditions and a quantitative method for long-term evaluation of the general thermal comfort conditions were added [21], they were only applicable to the mechanically conditioned buildings. To date, existing tools are designed to analyze and evaluate the unsteady thermal environment from the perspective of the building, an independent enclosed space [18], such as an office in a commercial building or a bedroom in a house, is usually the object of concern, rather than a human being who is truly exposed to different environments. In fact, a human can naturally and actively move between different rooms in a building, whether in an office building or a residential building. In addition, the human movements can also occur in buildings and outdoors. During these movements, people are exposed to the thermal environment in different spaces, which eventually forms a personal thermal environment experience. Especially, the personal environmental exposure to air temperature is called as personal experienced temperature. Therefore, the personal thermal environmental experience directly and validly reflects the actually thermal environmental conditions experienced by an individual and provides accurate insights into how often an individual encounters various conditions and how long exposures last, as well as when and why this occurs [22]. This makes it an important way to improve thermal comfort by continuously monitoring the personal thermal environment and studying its impacts on thermal comfort. This aids design of strategies to help people reduce uncomfortable experiences. In this way, continuous monitoring of the personal thermal environment and acquisition of basic data to support subsequent research has become a major priority.

Nevertheless, previous studies on the human thermal environment mainly focused on experiments in a controlled environment [23,24,25], point-in-time field tests [9,26], and fixed-point measurements [8,27,28]. These methods are desirable and valid for static humans, but for people moving around, it is necessary to select representative spatial samples for measurement [29], which obviously poses a great challenge to realistically record the environment to which people are exposed at each moment. On the other hand, there are many studies on human exposure in the field of public health, including pollutant exposure [30,31,32], occupational heat exposure [33,34], occupational cold exposure [34,35], and heat exposure[36,37]. In terms of thermal comfort, learning these methodologies for personal thermal exposure measurements seems worthwhile to try.

Therefore, on site measurements of personal experienced temperature for 14 persons in 6 different households in rural Beijing, China, were gathered in this study in order to better understand the personal thermal environment in rural areas of northern China. As an exploration, we find that using personal wearable monitors is a viable way to conduct similar research.

## 2. Methods and Materials

### 2.1. Research Site and Subjects

The authors conducted this pilot study in Erhezhuang village in southwestern peri-urban Beijing. This area was chosen because it was representative of northern China [5,38,39,40,41].

In this study, 6 eligible households were selected, and 14 permanent residences were recruited as test volunteers. Details are listed in the Table 1. The 14 volunteers constituted a balanced gender ratio. All the residents had an average age of 59.6 years old. All residents had lived in the village for a very long time, with the shortest residence time being 25 years. The people have fully adapted to the local climate and living habits, which objectively guaranteed that the research could reflect authentic situations. The 6 households were divided into 3 groups, and each group consisted of 2 households with same number of permanent residents. 

**Figure 1:**
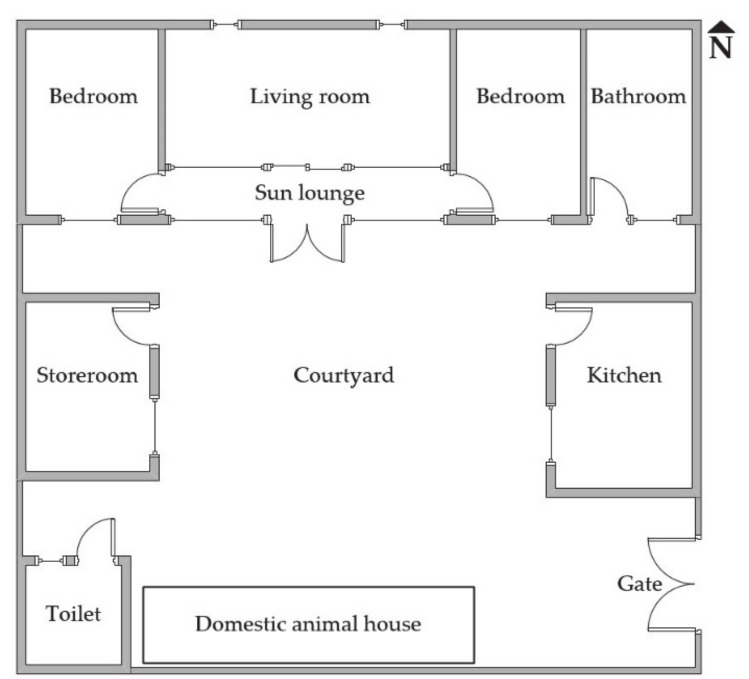
Typical floor plan of a rural residence in Beijing.

All 6 rural households were single-story buildings. A typical floor plan of a rural residential building in Beijing is shown in Figure 1. 

Unlike urban residential buildings, rural residences usually have an open courtyard and main rooms, including the living room, sun lounge, bedrooms, bathroom, storeroom, kitchen, toilet, and spaces for domestic animals, which are arranged around the courtyard. These zones, except the bedrooms and living room, are connected through an interior door or the sun lounge, while most of other functional zones are disconnected. If a resident wants to move from one functional zone to another, s/he has to pass through the courtyard. In addition, cleaning the courtyard, feeding domestic animals, and walking out after a meal are common habits. This means all people have to go in and out of rooms and are exposed to various environments naturally during their daily activities.

In these 6 households, conventional coal-burning stoves connected with circulating water radiators were used for space heating. During the coldest period, when coal-burning stoves supplied insufficient heat, some households also used split-type low temperature air-to-air heat pumps [3] for supplementary heating. 

During the on site study period, the volunteers can freely adapt their clothing and heating system as usual without any planned interventions from the study team.

### 2.2. Field Study Methods

The following methods were used to collect and obtain personal thermal environmental data.

#### 2.2.1. Continuous Field Test

A human being’s thermal sensation is influenced by clothing (or clothing insulation), physical activity (or metabolic rate), and various environmental parameters, including air temperature, air humidity, mean radiant temperature, and air velocity [18]. In order to obtain data for at least one full day or over multiple days without disturbing the volunteer’s daily behavior, a small portable monitoring instrument that could be carried by the volunteer was required. Due to lack of wearable instruments for testing the other four factors, only personal exposure to air temperature and humidity could be measured directly and accurately [22,37,42,43]. In fact, temperature was generally considered to be the most important environmental variable affecting thermal comfort [44]. Thus, as an exploration, volunteers’ experienced temperatures were examined to verify the feasibility of the proposed method, and wearable instruments were used to collect temperature data.

While different types of wearable devices have been successfully applied for specific tests, no single application guideline has dominated the others in all tests. Therefore, as a matter of exploration, the selection of instrument and the position where the volunteers wearing the instrument need to be carefully considered in this study.

**Figure 2:**
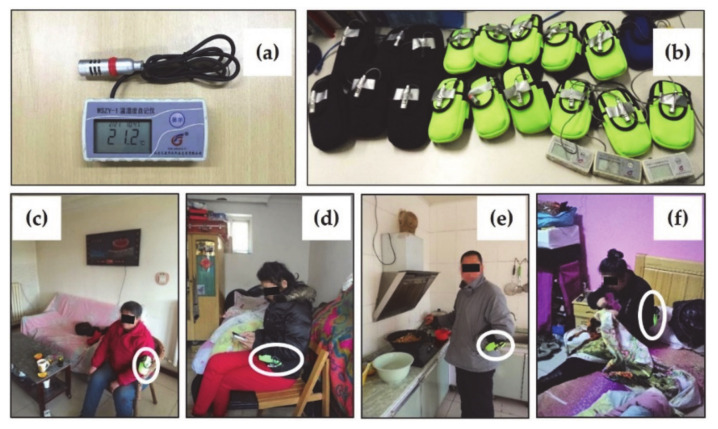
Personal experienced temperature monitoring devices. (**a**) Temperature logger and (**b**) devices used for monitoring. The three on the lower right were not placed in armband sleeves. (**c**) A volunteer wearing a device and sitting in the living room while watching TV. (**d**) A volunteer wearing a device and sitting in the bedroom playing with her smartphone. (**e**) A volunteer wearing a device and standing in the kitchen to cook. (**f**) A volunteer wearing a device and sitting in the bedroom to sew a quilt. The monitoring devices in (**c**–**f**) are circled with white ovals.

Firstly, as a device that needs to be adapted to daily wear, it should have at least the following features [45]: (i) easy-to-operate; (ii) energy efficient; (iii) miniaturized; (iv) light weight and portable; (v) well protected and robust; and (vi) capable of local data storage or wireless data transfer. Therefore, a temperature logger (WSZY-1B, Tianjian Hua Yi Co., Beijing, China), shown in Figure 2a, which possesses these features and also complies with the requirements of ISO 7726 [46], was selected for this study to continuously collect data. The instrument was small (98 mm × 49 mm × 25 mm) and light-weighted (~100 g), with a wide measurement range (−40 to 100°C), which can cover all possible extreme values, such as minimum outdoor temperature or maximum indoor temperature. The measurement accuracy was ±0.3°C and a thermal response time of 3 min to 90% in airflow of 1 m/s. A built-in rechargeable Li-ion battery provided enough power to collect 150,000 temperature data points. The main unit and sensor module were connected with a 0.75 m long wire.

Since the same device can obviously lead to different test results when worn at different locations on the body [47], uniformly specific location was considered in order to make the test results comparable. In existing studies, wearable devices were placed on the back [30], waist [24], wrist [48], hanging on the chest [44], clipped to the shoes [37], or placed into a carry-on backpack, and kept the probe outside [49]. Moreover, ISO 28802 recommends three different heights for similar tests: ankle, abdomen, and head [29]. Because this study attempted to continuously record volunteers’ daily temperature exposures, it was necessary to consider, as much as possible, the impact of the volunteers’ daily clothing and activity habits on device wear. For example, wearing the device on the head will inevitably affect the volunteers’ normal clothing, such as wearing a hat to keep out the cold; on the front chest or abdomen will be susceptible to the volunteers’ breathing airflow; on the back will affect the volunteers’ activities such as sitting or leaning on a sofa; on the legs will affect normal activities such as using the toilet; and on the ankles will be susceptible to effects such as trouser opening sloshing. Therefore, in order to obtain accurate data to reflect the environmental conditions of interest while minimizing the impact on volunteers’ normal activities as well as minimizing the impacts of human respiratory airflow and human thermal radiation on the measurement, this study finalized the placement of the temperature logger on the volunteer’s arm by communicating with the volunteers on site and trying it on.

Consequently, the sensor module was strapped onto a phone holder that was adapted as an armband sleeve, and the main unit was placed in the holder, as illustrated in Figure 2b. During the field test, the device (holder equipped with a logger) was worn by the volunteer, or it was placed near her/him close during special activities that required her/him to not wear an arm pocket, such as during sleep. Figure 2c–f shows the monitoring devices and some scenarios when volunteers wore the devices to participate in the test. For all measurements, instantaneous data were recorded with 1 min intervals. During the monitoring period, volunteers were also asked to record any time periods when they did not wear their monitors by pen and paper diary. 

**Figure A1:**
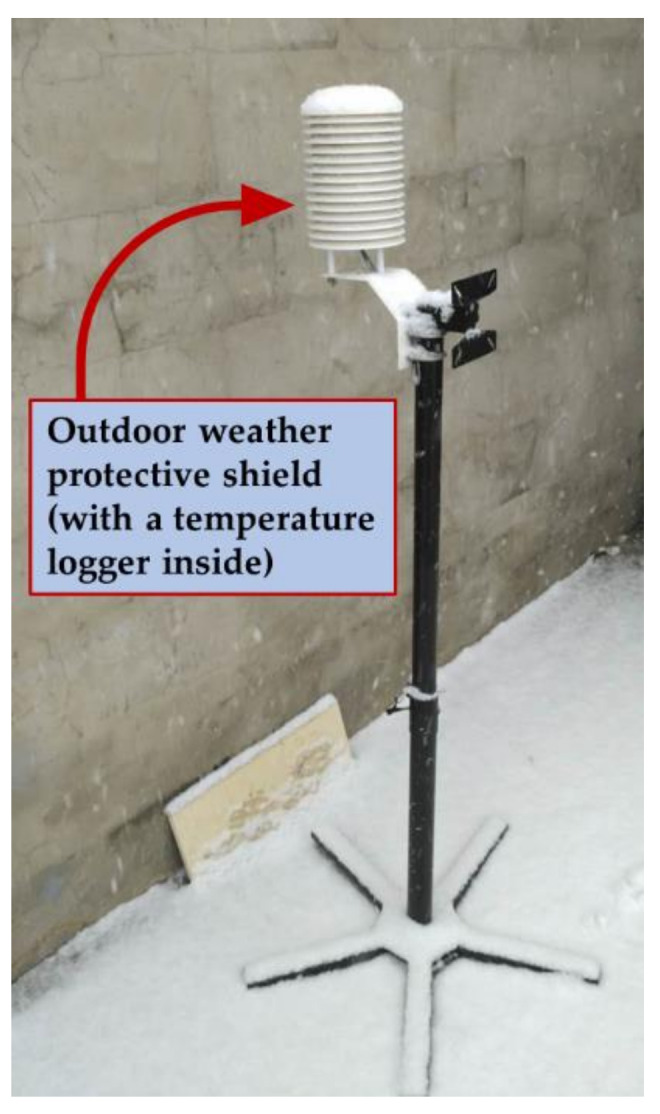
An outdoor air dry-bulb temperature logger with protection installed in household A#1.

As shown in Figure A1 of Appendix A, another temperature logger (WZY-1B, Tianjian Hua Yi Co., Beijing, China), with suitable range and precision (range: −40 to 60°C; ±0.3°C precision) as well as solar radiation, rain and snow protections was placed on the courtyard floor in household A#1 at 1.5 m height to record the outdoor dry-bulb temperature with 1-min interval.

#### 2.2.2. Questionnaire Surveys

In order to provide as comprehensive a picture as possible of the personal thermal exposure of the volunteers participated in this study in winter, information on the living habits, as well as the comfort and compliance of wearing the devices, and general thermal comfort of experienced thermal environments during the monitoring period, was gathered through a three-part questionnaire survey.

- Part A: Personal basic information and living habits in winter.
  In addition to the basic information of the volunteers listed in the Table 1 the ID number of the device given to each volunteer and the volunteers’ living habits in winter were recorded. Among the living habit questions related to adjusting clothing or altering the environment were listed in Table A1 of Appendix A.
- Part B: Personal thermal exposure experience.
  In the second part, we expected to gather information on personal thermal exposure experience during the monitoring period, while ensuring that the temperature data subsequently used for analysis was a valid record of the volunteer’s experienced temperature. For example, if a volunteer was outdoors but left the monitoring device indoors, temperature data recorded while the volunteer was not wearing the device as required would be eliminated prior to formal analysis. The volunteers had to respond to three questions regarding their habits while outside and four more questions primarily related to comfort and compliance of the monitoring devices (see Table A2 of Appendix A).
- Part C: General thermal comfort of experienced thermal environments.
  Along with obtaining test data on the volunteers’ experienced temperature, understanding their general thermal comfort with the thermal environment they experienced was one of the intended goals of the methods presented in this study. However, since the measurement did not cover all six parameters affecting thermal sensation, thermal comfort could not be analytical determined and interpreted directly according to the ISO 7730 [18] by calculating the PMV (predicted mean vote) and PPD (predicted percentage of dissatisfied) indices. It is well known that the use of subjective scales is an important tool for assessing thermal comfort of the physical environments. International standards ISO 10551 [50] and ISO 28802 [29] provide a number of subjective scales for thermal environment survey. However, the scales of these standards are mainly applicable to the point-in-time surveys and can only reflect the thermal sensation and comfort at the moment of survey. Therefore, it is clear from Table A3 of Appendix A that this study incorporated questions from both standards (e.g., questions C-5 and C-6 from ISO 10551, and question C-7 from ISO 28802) that were applicable to general thermal comfort surveys and developed four other questions following the principles of ISO 10551 to assess volunteers’ responses to the environment during the monitoring.

For each volunteer during the field investigation, Part A of the questionnaire should be answered only once, and it should generally be completed after the successful deployment of the wearable monitor. Parts B and C should be carried out in pairs each time, and the first time should be completed on the second day after the deployment of the monitor, followed by 1–2 days to carry out once, not only in order not to overly interrupt the normal life of the volunteer, but also to avoid confusing the volunteer by recalling an overlong experience. The questionnaires for the above three parts were printed before the field visit. All questionnaires were filled out by an appointed investigator, who was responsible for posing questions to the volunteers, recording the answers, and responding to questions raised by the volunteers during the survey period.

### 2.3. Time Period of the Field Investigation

As a pilot study, the actual thermal environment and thermal comfort of the residents under the worst conditions were mostly concerned, for instance, during the coldest period. In addition, conducting research during the coldest period helps to verify the feasibility of the proposed method. Therefore, weather forecasts were used to deliberately select the period from January 22nd to 25th, 2016, for field investigation. This period belonged to an extreme cold wave period [51] and provided a valuable opportunity to capture data regarding the actual thermal exposure of rural residents during the coldest period in winter. During this period, all monitors were deployed and started on the 22nd while each volunteer completed a survey including Part A above. Monitoring continued and a survey including Parts B and C was conducted on the 23rd. Monitoring continued on the 24th. The monitoring ended and the last survey for each volunteer including Parts B and C was conducted on the 25th. Prior to the field investigation, this study protocol was determined to comply with and be approved by the ethical review board of Tsinghua University (20140077). Informed consent was obtained from all volunteers for this study.

### 2.4. Data Analysis

#### 2.4.1. Data Processing and Data Analysis

All descriptive and statistical analyses of the field and survey data were performed in Microsoft Excel and OriginPro 2017 (b9.4.0.220, OriginLab Co., Northampton, MA, USA). An analysis of variance (ANOVA) test was used in this study to assess differences between the volunteers’ personal experienced temperatures. 

#### 2.4.2. Reference Scenarios for Analyses

To describe the volunteers’ experienced thermal environments and comfort levels, the recommended indoor temperatures specified in the three current criteria in Table 2 were used as reference scenarios. As important precedent conditions, clothing assumptions are also listed in the table. In ASHRAE Standard 55-2017 [52], the comfort band is defined as conditions where the PPD people reaches 10%, corresponding PMV levels from –0.5 to +0.5. Therefore, the stated boundary for the temperature comfort zone in winter ranged from 19.6 to 25.7 °C. In contrast to the ASHRAE standard, the comfort zone was defined as conditions of PPD not exceeding 25% and the PMV levels ranging from –1 to 0 in the Chinese National Standard GB 50736-2012 [53], which are based on energy benefits. Consequently, the upper and lower boundaries for the temperature comfort zone were 18 and 24 °C, respectively, which are lower than those in the ASHRAE standard. This range is quite reasonable for buildings in urban areas of China. However, in rural areas, houses are usually poorly insulated and are not air tight. Therefore, most residents have adapted to cold temperatures in winter. Through many large-scale studies in rural areas [38], the temperature comfort zone starts at 10 °C and ends at 18 °C, which was specifically suitable for rural areas in China, as defined in the CECS (China Association for Engineering Construction Standardization) Standard 332:2012 standard [54].

## 3. Results

### 3.1. Survey Results on Living Habits in Winter and the Thermal Environment Experiences

#### 3.1.1. Personal Living Habits in Winter

**Figure 3:**
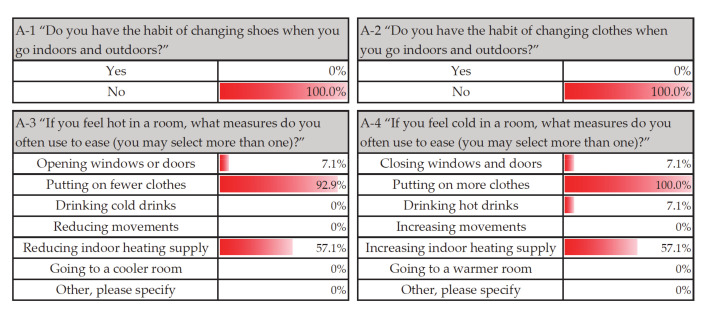
Personal living habits in winter of volunteers (N = 14).

All 14 volunteers participated in the first part (Part A) of the survey; their personal living habits in winter are shown in Figure 3. In questions A-1 and A-2, nobody changed shoes or clothes when going indoors or outdoors, which is typical of urban residents in the same region in winter [2]. Questions A-3 and A-4 yielded similar responses. Regarding the most uncomfortable thermal situations, almost all people (92.9% and 100%, respectively) chose to change clothes as adjustment measures. The same 8 out of 14 volunteers (57.1%) also adjusted their heating system, e.g., banking/poking up the coal stove or adjusting the desired temperature of their low temperature air-to-air heat pumps. Only one (7.1%) chose to open/close doors or windows, and another chose to consume hot drinks. Consequently, clothing is the first and most important adjustment measure for volunteers, which is another typical living habit in winter.

#### 3.1.2. Personal Thermal Exposure Experience

The survey results are summarized in Table 3. Regarding the outside environment addressed in question B-1, all residents (100% and 85.7%, respectively) indicated they went outdoors except for two volunteers (P#7 and P#8 in household C#1), who stayed indoors on the 24th due to physical reasons. In response to question B-2, none of the volunteers changed their shoes when they went indoors or outdoors. In response to question B-3, none of the volunteers changed their clothes because of outdoor domestic work rather than thermal comfort except for P#10, who changed his daily coat to his housework coat. All volunteers provided negative answers to questions B-4 to B-6 in both surveys, indicating the residents accepted and adapted to the proposed test method. This also shows that the obtained data using this method can represent the actual environmental exposure of volunteers. In response to question B-7, all volunteers removed the device while sleeping and placed it on the bedside table or a small tea table in the bedroom as they were instructed. This period of time generally began around 21:00 and ended around 7:00 every day, based on the records noted by volunteers. 

### 3.2. Overview of Outdoor air Temperature and Personal Experienced Temperatures

**Figure 4:**
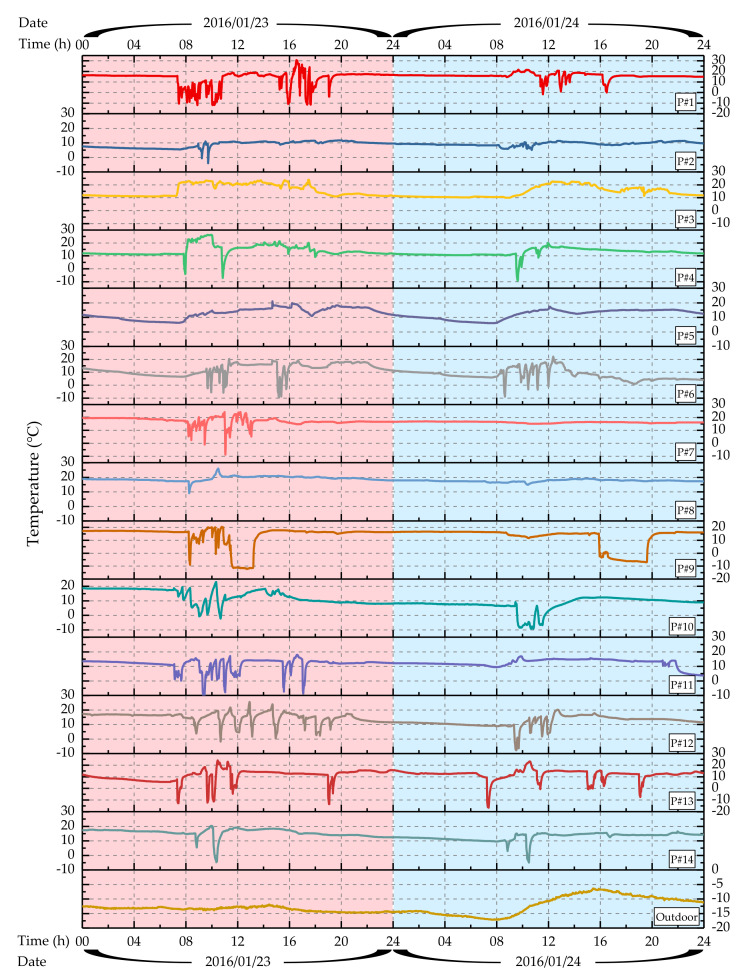
Outdoor air temperature and 14 volunteers’ personal experienced temperatures from January 23rd to 24th, 2016. P#1–14 is the volunteers’ ID number, which coincides with the information in Table 1.

The devices successfully monitored and recorded personal experienced temperatures throughout the field study period. Because each volunteer wore the devices for different time periods, outdoor air temperature data and volunteers’ personal experienced temperature from 00:00 on January 23rd to 24:00 on the 24th, 2016, was selected, as shown in Figure 4. Throughout the period, the outdoor air dry-bulb temperature ranged from −17.1 to −6.4 °C, with an average of −12.7 °C. These temperatures are much lower than the average temperature of −0.7 °C during the past 30 winters and the historical extreme minimum temperature of −18.3 °C [53]. 

The following points can be observed from the figure.

- Volunteers had different and individualized personal temperature experiences.

In the figure, the temperature curves for different volunteers are very different. Each curve has its own individual temperature changes, and experiences during the first and second days were different for the same volunteer.

- Temperature changes during daytime are distinct from those at night.

Basically, the period containing obvious temperature changes was mainly concentrated from 7:00–21:00. This coincides with the details of volunteers’ lifestyle habits during the survey. They often went to bed after 21:00 and got up at around 7 o’clock the next morning, and few outdoor activities occurred during this period. Therefore, one day was divided into daytime (7:00–21:00) and nighttime (21:00–7:00) periods.

### 3.3. Personal Experienced Temperature Ranges and Distribution

**Figure 5:**
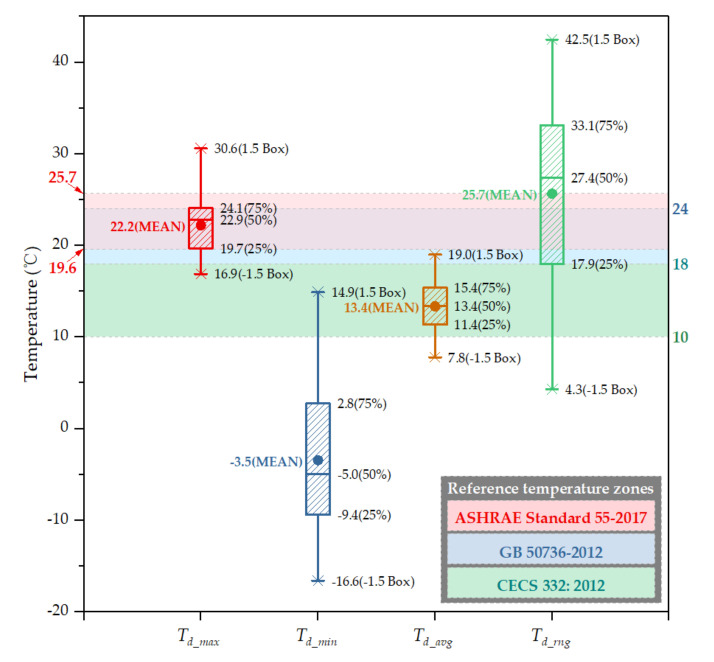
Statistical results of daily personal experienced temperature.

The statistical data on the daily maximum *T_d_max_*, daily minimum *T_d_min_*, daily average *T_d_avg_*, and diurnal range (temperature difference) *T_d_rng_* in the personal experienced temperature for 14 persons for two days are summarized as Figure 5. The *T_d_max_* of 14 volunteers experienced ranging from 16.9 to 30.6 °C with a mean value of 22.2 °C. Most of the temperature data (64.3%) were within the union of comfort temperature ranges defined in ASHRAE Standard 55-2017 and GB 50736-2012. The *T_d_min_* ranged from −16.6 to 14.9 °C with a mean value as low as −3.5 °C. Most of the data (89.3%) were lower than the three reference standards, i.e., below 10 °C. Therefore, most of the *T_d_avg_* data (82.1%) fell into the reference temperature band specified in CECS 332:2012 with a large *T_d_rng_*, while the proportion of data greater than over 10, 20, and 30 °C was 96.4%, 71.4%, and 35.7%, respectively. Moreover, the maximum *T_d_rng_* exceeded 40 °C, up to 42.5 °C, and the mean *T_d_rng_* was 25.7 °C. However, in the twice evaluations, all volunteers indicated that there were no uncomfortable or unacceptable air temperatures during the study period, volunteers unanimously accepted the thermal environment they experienced (see Table 4), including multiple temperature transients and the large diurnal temperature range. Consequently, such personal temperatures appear to be reasonable.

**Figure A2:**
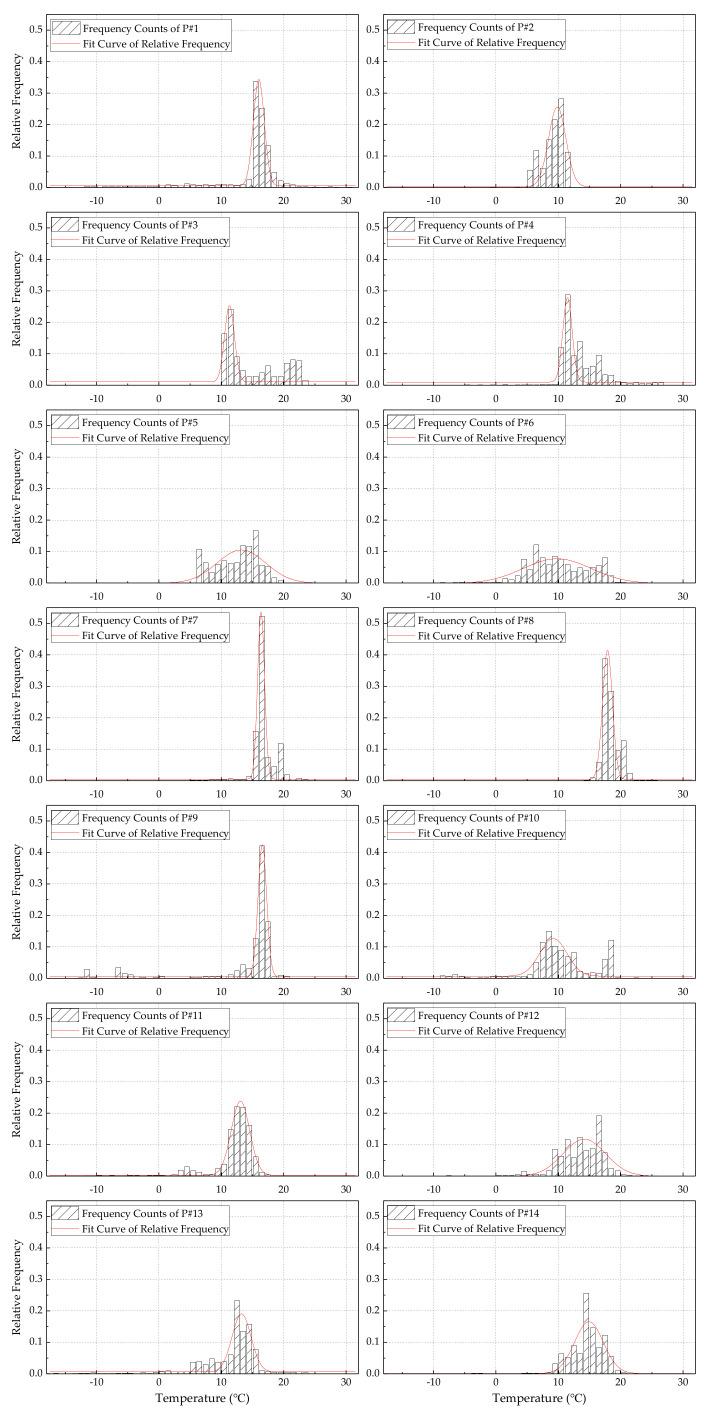
Personal experienced temperature distributions from the 14 volunteers. P#1–14 is the volunteers’ ID number and matches the ID in Table 1.

The personal experienced temperature distributions for the 14 volunteers are shown in Figure A2 of Appendix A. Furthermore, the adjusted *R^2^* values range from 0.66 to 0.98 in Table 5, indicating that these distributions likely followed a Gaussian distribution. However, the distributions varied somewhat. The average temperature ranged from 9.22 to 17.89 °C, and most average values fell within the comfort zones specified in CECS 332:2012. The average temperatures experienced by four volunteers (P#1, P#7, P#8, and P#9) were greater 16 °C and had standard deviation ranging from 0.56 to 0.88 °C, indicating that the personal experienced temperature distribution is more concentrated. In contrast to this, the average temperatures experienced by three volunteers (P#2, P#6, and P#10) were lesser than 10 °C and had a higher standard deviation ranging from 1.39 to 5.45 °C, which meant that their personal experienced temperatures were more dispersed.

### 3.4. Differences in Personal Experienced Temperatures

**Figure 6:**
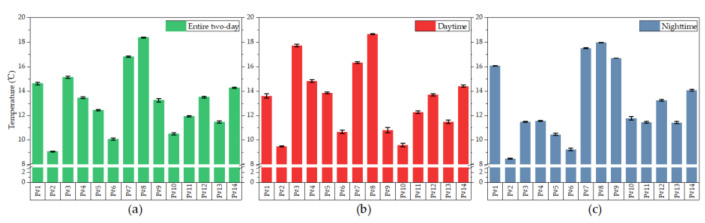
Personal mean experienced temperature with standard error. Personal mean experienced temperature (**a**) over the entire two-day period, (**b**) during daytime, and (**c**) during nighttime.

**Figure 7:**
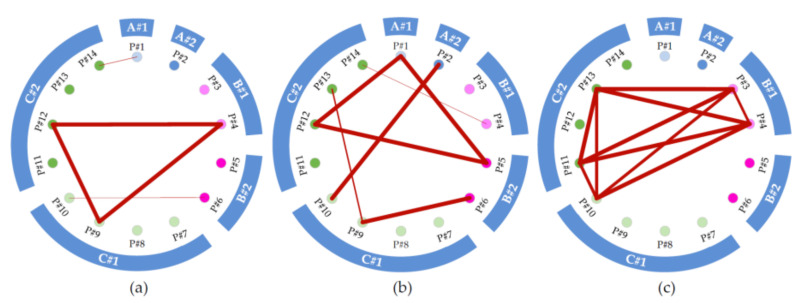
Pairs with no significant differences between mean personal experienced temperatures. (**a**) Pairs throughout the entire two-day period; (**b**) pairs during daytime; (**c**) pairs during nighttime. Crimson lines indicate that the connected volunteers experienced mean personal temperatures that were not significantly different. A thicker line indicates a larger *p*-value.

The results in Section 3.1 show that the volunteers have similar living habits in winter and during the test period, but the personal data in Section 3.2 and Section 3.3 were different. To assess their differences, we used ANOVA to compare the personal experienced temperature data of 14 volunteers and tested our initial hypothesis that the personal temperature variance was equal for all volunteers. However, this homogeneity assumption of equal variances was rejected at the 0.05 level using Levene’s test. Further, we used the Welch test to examine differences in the mean personal experienced temperature between volunteers, and the results show that the population means are significantly different at the 0.05 level. This means that at least two of the 14 volunteers have a different mean experienced temperature. To determine exactly which mean differences are significant and which are not, the Games–Howell test, a post hoc test without assumed equal variance was used to compare the mean temperatures experienced by the 14 volunteers. The test was applied to the data from the daytime and nighttime data, as well to data for the entire two-day period. Figure 6 shows the mean personal experienced temperature with standard error, and Figure 7 shows pairs whose difference in means are not significant in the three tests, where the *p*-values are greater than the significance level of 0.05.

In the three tests, more than half the volunteers experienced a different temperature in a statistical sense. Throughout the two-day period (see Figure 6a and Figure 7a), P#1 and P#14 are paired and statistically experienced the same temperature level (approximately 14.5 °C). P#4, P#9, and P#12 are paired and experienced the same temperature (approximately 13.4 °C). P#6 and P#10 form another pair and experienced the same temperature (approximately 10.3 °C). The remaining 7 volunteers experienced statistically different temperatures, ranging from 9.1 to 18.4 °C. Results from the other two tests have similar interpretations. It is worth noting that volunteers who experienced the same temperature throughout the two-day period did not necessarily experience the same temperature in daytime or nighttime, and vice versa. A study by Kuras and Hondula [43] found that the participants’ experienced temperatures were significantly different, even though they were from the same neighborhood in Boston. Furthermore, in our study, we find significant differences among temperatures experienced by the participants, even though they were from the same family and lived together for decades. For example, volunteers P#7, P#8, P#9, and P#10 from household C#1 experienced significantly different temperatures, and no counter-example can be found in all three tests. This strengthens one of our findings, namely, that similar living habits and behaviors do not necessarily result in two different people experiencing the same temperature. 

## 4. Discussion 

### 4.1. Continuous Field Test

As an exploratory study, there are some limitations that should be considered in the future. It is worth considering expanding the scope of the study by including more measurements of the environmental parameters of the space experienced by the volunteers. Indeed, we have attempted to do so. However, measuring them is difficult. First, volunteers may go to different spaces every day, such as different rooms in their own house, walk to visit their neighbors’ houses in the same village, or take transportation to places farther away from the village. Moreover, these space experiences that follow daily routines and habits may be different every day, with strong randomness and independence [55]. Therefore, it is very difficult to deploy measurements for each space they have occupied. Furthermore, even if environmental information on each space experienced by the volunteers was somehow tested and obtained, it would not be possible to confirm when and how long the volunteers occupied each particular space, as it is realistically difficult to obtain precise information on the location of the volunteers at each moment. It is therefore difficult to rigorously correlate the environmental information with the volunteers’ individual exposure experiences. This paper therefore focuses as an exploratory study on efforts to address the testing of personal experienced temperature exposures. Nevertheless, this consideration is a very meaningful direction and could be one of the valuable directions for future studies with the prospect of success.

There are six factors that influence thermal comfort. When these factors are measured, the thermal sensation and comfort can be predicted by calculating the PMV. However, constrained from experimental reality, as the lack of wearable instruments can be used to measure all of these factors, the influence of factors other than air temperature were not continuously monitored. Hence, long-term continuous evaluation of the general thermal comfort during field investigation could not be fully conducted. As an alternative, a survey was used to record participants’ evaluation for the general thermal sensation and comfort. The 14 volunteers provided similar qualitative results in twice studies, so it was not easy to compare their different thermal experiences based solely on qualitative evaluation. More refined studies on this matter should be conducted by considering the development of other factor synchronous testing, or perhaps by conducting a more in-depth quantitative evaluation of the general thermal environment.

Important in all cases, it is recommended that calibration be performed both before and after testing to check for any temperature “drift”. The data presented in this article was completely reliable based on two instrument calibrations performed by the manufacturer both before and after the field test.

### 4.2. Questionnaire Survey

As indicated previously, the questions in the ISO standards are not fully applicable to the long-term general thermal environment investigation in this study, so new suitable questions need to be developed based on the incorporation of some of the applicable questions in the existing standards. In all cases, however, the principles of the ISO standard for constructing questions and procedures for administering them should be followed. 

In this study, three subjective questions on physical ambience such as personal tolerance, acceptability, and satisfaction were incorporated from the standards ISO 10551 [50] and ISO 28802 [29], as shown in questions C-5 to C-7 in Table A3 of Appendix A, while questions on personal state such as perception, evaluation, and preference, which are more suitable for the point-in-time survey, were discarded. Moreover, the four questions (C-1 to C-4) on air temperature and general thermal environment experience were set before questions C-5 to C-7 in order to provide a basis for the overall judgment related to the latter three questions.

It is a very practical approach to carry out a pre-survey on site prior to the formal survey so that questions and response options can be optimized. For example, in the response options for question C-5, a continuous and gradual scale of “tolerable, slightly difficult to tolerate, fairly difficult to tolerate, very difficult to tolerate, intolerable” was used, whereas the pre-survey found that in order to answer this question appropriately, residents often fell into the trap of searching for the details of various thermal environment they had experienced over a period of time, rather than giving an “overall” rating. Therefore, in order to reduce bias and ensure validity and reliability of response, this study optimized the options to two-category statement. Similar questions such as C-6 and C-7 were optimized similarly to provide consistency in the questions of valid and reliable scales.

The survey results in Section 3.1 show that changing clothes was the preferred method for adjusting to uncomfortable situations, but the volunteers had not changed their clothes for thermal comfort reasons during the study period. The reason participants could not wear more clothing may have been due to the monitoring equipment on their arm that prevented them from changing clothing, therefore, three questions (B-4 to B-6 in Table A2 of Appendix A) were set to ensure that the data analyzed subsequently were not affected by this situation (if this was the case, the corresponding data would be eliminated prior to formal analysis). 

A favorable relationship between the investigators and the participants is beneficial for obtaining valid results [50], especially in surveys that are not anonymous. One of the reasons why the village of Erhezhuang was chosen for this pilot study was because the research team had previously conducted research in this village for over ten years and we had developed a good relationship with the residents, thus ensuring that the participants in this study answered the relevant questions freely and honestly.

### 4.3. Data Analysis and Interpretation

Volunteers experienced varying degrees of low temperatures during the testing period, yet 14 out of 14 volunteers never found the temperature or thermal environment unacceptable or uncomfortable, and were generally tolerant, acceptable, and satisfied with the thermal environment they experienced. This may seem like a surprising result, but the residents who participated in our study followed similar habits and gave the same subjective judgments during follow-up visits over the next two heating seasons. Therefore, we consider the results of the study to be reliable. However, it should be noted with caution that thermal environment is acceptable does not necessarily mean it is preferable. 

Moreover, the volunteers accept the low temperatures thanks also to the high clothing insulation level. They were also asked to describe their clothing in the present study. During the study period, thermal resistance of the clothing worn by conscious volunteers ranged from 1.15 clo and 2.17 clo, with an average of 1.62 clo. This exceeds the 1.00 clo clothing assumption in Table 2 and was nearly twice as large as the average clothing insulation of 0.83 clo [56] in urban areas of Beijing in heating season. On the one hand, the large thermal resistance may explain why residents can protect themselves against cold during short-term outdoor activities in winter. On the other hand, the fact that volunteers did not change their clothes can be regarded as evidence that volunteers did not experience any discomfort. However, it is noted that the maximum clothing insulation was roughly twice as large as the minimum between the 14 volunteers. Different clothing insulation obviously affects thermal comfort, but a strict comparison of thermal sensation and comfort with different levels of insulation from clothing was not investigated. A comparison will require larger and longer-term studies in the future.

As an exploration, the results of the field test presented in Section 3 have sufficiently demonstrated the feasibility and validity of the method proposed in this paper. Limited by the major goal and length of the present study, more worthwhile in-depth data analyses such as thermal response time and uncertainty analysis, temperature fluctuation and instantaneous rate of change, personal experienced temperature compared to the outdoor temperature, and the effect of thermal experience on thermal environment expectations, as well as the specific effects of different exposures on thermal sensation and comfort will be discussed in separate articles.

As a new perspective on understanding the actual thermal comfort and heating demands of rural residents in winter, the main purpose to monitor and collect data on residents’ thermal environmental experiences is to seek out their personal uncomfortable experiences and to help them reduce these experiences and improve their thermal comfort during the heating season. In the context of the clean energy heating action in China, to meet the personal heating demands of different rooms and members of the rural households could lead to a win–win situation (maintaining personalized thermal comfort while saving heating energy). As a direct outcome, and combining with our previous research experiences [3,4,12,38,39], individual equipment to heat rooms individually and separately, rather than the conventional centralized-heating that is difficult to control individual demands could be suggested. This proposal has been well implemented in China rural households, where millions of individual room-based air source heat pumps have been utilized in the past three years [2].

## 5. Conclusions

People are exposed to a variety of thermal environments that can affect their comfort and health every day. In this study, we introduced the air temperature exposure test method to continuously gather field measurements of personal temperatures experienced in a particular environment. In total, 14 permanent residents in 6 different households from the same village in rural Beijing, China, were recruited to participate in the experiment. The personal temperature experienced by each volunteer was captured after monitoring and gathering survey responses during an extreme cold wave. These results lead to the following conclusions:

- The personal experienced temperatures gathered during the two-day period exhibit wide ranges and large variations. Meanwhile, the highest and lowest temperatures experienced by the residents reached 30.6 and −16.6 °C, respectively.
- The residents had very similar or identical living habits in winter and coping strategies for thermal discomfort, as well as same general thermal comfort evaluation results for experienced thermal environment during the study period. However, there were some obvious differences in the personal temperatures experienced by different households from the same village, and even between different residents from the same household. This suggests that similar or identical living habits, coupled with same general thermal comfort level cannot infer that residents are at the same level of thermal environment.
- As far as methodology is concerned, this study indicates that using personal wearable monitors is a viable method to measure the temperature experienced by an individual. It not only extended the method of collecting data regarding complex experiences in different environments, but also could help in the study of human responses to the real-world thermal environment.

## Figures and Tables

**Table 1:** Information on the 6 households and 14 permanent residents.

Group	Household ID	Permanent Population	Permanent Residents’ ID	Gender	Age	Years of Living Locally
A	A#1	1	P#1	Female	51	51
	A#2	1	P#2	Male	80	80
B	B#1	2	P#3	Female	47	47
			P#4	Male	49	49
	B#2	2	P#5	Female	58	58
			P#6	Male	61	61
C	C#1	4	P#7	Female	46	46
			P#8	Female	70	70
			P#9	Male	47	25
			P#10	Male	73	73
	C#2	4	P#11	Female	51	51
			P#12	Female	74	74
			P#13	Male	49	49
			P#14	Male	78	54

**Table 2:** Recommended indoor temperature and assumed conditions for winter (heating season) in three standards.

Standard	PPD ^1^ (%)	PMV ^2^ Range	Indoor Temperature (°C)	Clothing Assumption (clo)
ASHRAE ^3^ 55-2017 [52]	<10	−0.5 < PMV < +0.5	19.6–25.7	1.0
GB ^4^ 50736-2012 [53]	≤25	−1 ≤ PMV ≤ 0	18–24	1.0
CECS ^5^ 332:2012 [54]	—	—	10–18 ^6^	1.2–2.0 ^7^

^1^ PPD: predicted percentage dissatisfied. ^2^ PMV: predicted mean vote. ^3^ ASHRAE: American Society of Heating, Refrigerating and Air-Conditioning Engineers. ^4^ GB: Chinese National Standard. ^5^ CECS: China Association for Engineering Construction Standardization. ^6^ This preferred temperature range was determined through extensive field studies over the years and was not determined with the PMV method. ^7^ This range is not an assumption, rather it is a range with high probability that was calculated based on real clothing worn during winter.

**Table 3:** Responses to questions in the “personal thermal exposure experience” part (N = 14).

Questions	Answer Options	Selection Results	
		1st Survey	2nd Survey
B-1 “Have you ever been to the outside since wearing the device or since our last visit?”	-Yes -No	14 0	12 2
B-2 “Have you changed your shoes when you go indoors and outdoors since wearing the device or since our last visit?”	-Yes, please specify -No	0 14	0 14
B-3 “Have you changed your clothes when you go indoors and outdoors since wearing the device or since our last visit?”	-Yes, please specify -No	0 14	1 13
B-4 “Do you have any trouble or feeling uncomfortable by wearing the device?”	-Yes, please specify -No	0 14	0 14
B-5 “Does wearing the device affect your normal movements or lifestyle?”	-Yes, please specify -No	0 14	0 14
B-6 “Have you deliberately changed your behavior because of wearing the device?”	-Yes, please specify -No	0 14	0 14
B-7 “According to your records, please indicate the (period of) time when you did not wear the device since the first time you put it on or since our last visit (you may select more than one option)?”	-Bedtime at night -Naptime in the afternoon -Other, please specify	14 0 0	14 0 0

**Table 4:** General thermal comfort as indicated by participants (N = 14).

Questions	Answer Options	Selection Results	
		1st Survey	2nd Survey
C-1 “Have you ever felt uncomfortable with the air temperature since wearing the device or since our last visit?”	-Yes, please specify -No	0 14	0 14
C-2 “Have you experienced any unacceptable air temperature since wearing the device or since our last visit?”	-Yes, please specify -No	0 14	0 14
C-3 “Have you ever felt uncomfortable with the general thermal environment since wearing the device or since our last visit?”	-Yes, please specify -No	0 14	0 14
C-4 “Have you experienced any unacceptable thermal environment since wearing the device or since our last visit?”	-Yes, please specify -No	0 14	0 14
C-5 “In your opinion the thermal environment you have experienced since wearing the device or since our last visit is tolerable?”	-Yes -No	14 0	14 0
C-6 “Taking into account your personal preference only, would you accept rather than reject the thermal environment you have experienced since wearing the device or since our last visit?”	-Yes -No	14 0	14 0
C-7 “Are you generally satisfied with thermal environment you have experienced since wearing the device or since our last visit?”	-Yes -No	14 0	14 0

**Table 5:** Personal experienced temperature distribution for each volunteer.

Permanent Residents’ ID	Distribution Type and Formula	*μ*		*w*		Adjusted *R^2^* ^2^
		Value	*S.E.* ^1^	Value	*S.E.*	
P#1	Distribution type: Gaussian distribution The specific formula described below was used for the fitting. $y=y_{0}+\frac{A}{w\sqrt{\pi /2}}e^{-2\frac{{\left(x-\mu \right)}^{2}}{w^{2}}}$ where *y_0_* is the baseline offset, *μ* is the mathematical expectation of the Gaussian distribution, *w* equals 2 times the standard deviation of the distribution or approximately 0.849 the width of the peak at half height, and A is the area under the peak of Gaussian distribution curve.	16.01	0.05	1.77	0.10	0.93
P#2		9.84	0.10	2.78	0.21	0.87
P#3		11.31	0.09	1.59	0.17	0.76
P#4		11.48	0.09	1.50	0.16	0.73
P#5		13.22	0.41	7.81	0.92	0.72
P#6		9.82	0.49	10.90	1.18	0.75
P#7		16.38	0.04	1.11	0.05	0.95
P#8		17.89	0.05	1.58	0.11	0.91
P#9		16.58	0.02	1.38	0.04	0.98
P#10		9.22	0.28	4.35	0.60	0.66
P#11		13.07	0.04	2.96	0.08	0.98
P#12		14.18	0.33	6.84	0.72	0.76
P#13		13.25	0.13	3.05	0.26	0.84
P#14		14.91	0.20	4.60	0.43	0.81

^1^*S.E.*: standard error. ^2^*R^2^*: coefficient of determination.

## Appendix A

**Table A1:** Summary of questions in the “personal basic information and living habits in winter” survey.

Questions	Answers
A-1 “Do you have the habit of changing shoes when you go indoors and outdoors?”	-Yes -No
A-2 “Do you have the habit of changing clothes when you go indoors and outdoors?”	-Yes -No
A-3 “If you feel hot in a room, what measures do you often use to ease (you may select more than one)?”	-Opening windows or doors -Putting on fewer clothes -Drinking cold drinks -Reducing movements -Reducing indoor heating supply -Going to a cooler room -Other, please specify
A-4 “If you feel cold in a room, what measures do you often use to ease (you may select more than one)?”	-Closing windows and doors -Putting on more clothes -Drinking hot drinks -Increasing movements -Increasing indoor heating supply -Going to a warmer room -Other, please specify

**Table A2:** Summary of questions in the “personal thermal exposure experience” survey.

Questions	Answers
B-1 “Have you ever been to the outside since wearing the device or since our last visit?”	-Yes -No
B-2 “Have you changed your shoes when you go indoors and outdoors since wearing the device or since our last visit?”	-Yes, please specify -No
B-3 “Have you changed your clothes when you go indoors and outdoors since wearing the device or since our last visit?”	-Yes, please specify -No
B-4 “Do you have any trouble or feeling uncomfortable by wearing the device?”	-Yes, please specify -No
B-5 “Does wearing the device affect your normal movements or lifestyle?”	-Yes, please specify -No
B-6 “Have you deliberately changed your behavior because of wearing the device?”	-Yes, please specify -No
B-7 “According to your records, please indicate the (period of) time when you did not wear the device since the first time you put it on or since our last visit (you may select more than one option)?”	-Bedtime at night -Naptime in the afternoon -Other, please specify

**Table A3:** Summary of questions in the “general thermal comfort of experienced thermal environments” survey.

Questions	Answers
C-1 “Have you ever felt uncomfortable with the air temperature since wearing the device or since our last visit?”	-Yes, please specify -No
C-2 “Have you experienced any unacceptable air temperature since wearing the device or since our last visit?”	-Yes, please specify -No
C-3 “Have you ever felt uncomfortable with the general thermal environment since wearing the device or since our last visit?”	-Yes, please specify -No
C-4 “Have you experienced any unacceptable thermal environment since wearing the device or since our last visit?”	-Yes, please specify -No
C-5 “In your opinion the thermal environment you have experienced since wearing the device or since our last visit is tolerable?”	-Yes -No
C-6 “Taking into account your personal preference only, would you accept rather than reject the thermal environment you have experienced since wearing the device or since our last visit?”	-Yes -No
C-7 “Are you generally satisfied with thermal environment you have experienced since wearing the device or since our last visit?”	-Yes -No
